# Impact of evidence-based nursing intervention based on Watson’s theory on rehabilitation compliance in elderly patients after intertrochanteric femur fracture surgery

**DOI:** 10.3389/fmed.2026.1714184

**Published:** 2026-05-26

**Authors:** Kun Zhang, Jie Chen, Yaqiong Cao, Jie Li, Chen Huang, Yanhong Zhu

**Affiliations:** Department of Orthopedics, The Affiliated Hospital of Xuzhou Medical University, Xuzhou, Jiangsu, China

**Keywords:** evidence-based nursing, intertrochanteric femur fracture, postoperative pain, postoperative recovery, rehabilitation compliance, Watson theory

## Abstract

**Objective:**

The study aimed to unveil the impact of evidence-based nursing (EBN) based on Watson’s theory on rehabilitation compliance in elderly patients with intertrochanteric femur fractures.

**Methods:**

A prospective randomized controlled trial design was adopted. Eighty elderly patients with intertrochanteric femur fractures were randomly divided into a control group (received routine intervention), and an observation group (received EBN intervention based on Watson’s theory), with 40 cases in each group. Pain levels were assessed using the Visual Analog Scale (VAS) at 3 days, 7 days, and 1 month post-surgery. Postoperative outcomes, including weight-bearing walking time, fracture healing time, and hospital stay, were compared. Quality of life was evaluated using the WHOQOL-BREF. Rehabilitation adherence was assessed based on training content, duration, intensity, initiative, and completion. Patient satisfaction with care was measured using a custom satisfaction survey. Harris Hip Score (HHS) was adopted to assess joint function recovery. Complications were recorded for both groups.

**Results:**

The observation group had significantly lower VAS scores (*P* < 0.01), shorter weight-bearing walking time, fracture healing time, and hospital stay (*P* < 0.01). The observation group showed higher WHOQOL-BREF scores, better rehabilitation adherence, higher satisfaction, and higher HHS (*P* < 0.01). Besides, the observation group had fewer complications (*P* < 0.01).

**Conclusion:**

EBN based on Watson’s theory in elderly patients with intertrochanteric femur fractures can reduce postoperative pain and complications, improve hip joint function, enhance quality of life, and result in better postoperative recovery, with overall significant effectiveness.

## Introduction

Intertrochanteric fractures are prevalent, constituting nearly half of all hip fractures, and impose a significant strain on orthopedic healthcare services (1). These fractures are characterized as extracapsular fractures of the proximal femur, occurring between the greater and lesser trochanters (2). Trochanteric fractures require significant medical and surgical intervention, more so than many other fractures. Unlike adults, children recover better due to higher osteoblastic activity and better blood supply to the femoral head. Older individuals, however, are more commonly affected by such trauma (3). Intertrochanteric femur fractures are linked to high morbidity/mortality, requiring approaches that minimize anesthesia time, blood loss, and further injury, all while ensuring optimal fixation in osteoporotic bone (4). As the problem of postoperative rehabilitation compliance seriously affects the recovery process of patients, improving the rehabilitation compliance of elderly postoperative patients with intertrochanteric femur fractures has become an important task in clinical nursing.

Evidence-based nursing (EBN) is a care model that prioritizes the integration of scientific research with the creation of personalized care plans tailored to a patient’s specific condition (5). As EBN research progresses, nursing practices are increasingly grounded in scientifically reliable evidence from patient outcomes. This shift is transforming the previously narrow, empiricist model of nursing into a more modern and conceptually advanced approach (6). The human caring theory framework is a cornerstone of the nursing discipline (7). Watson (8) adopted the World Health Organization definition of health, which includes physical, mental, and social wellbeing, and the ability to engage in daily activities. Her theory aligns with modern strategies to improve the health of individuals and families, and can be applied to the care of patients’ caregivers (9). Therefore, EBN interventions based on Watson’s theory can promote patients’ understanding and acceptance of the rehabilitation program through careful nursing measures and good communication mechanisms, thus achieving better rehabilitation outcomes.

Although existing literature suggests the potential value of nursing based on Watson’s theory (10, 11), research on its specific effects on rehabilitation compliance in elderly patients after intertrochanteric femur fracture surgery remains limited. Therefore, the primary objective of this study is to explore the impact of EBN intervention based on Watson’s theory on rehabilitation compliance in elderly patients after femoral intertrochanteric fracture surgery. The secondary objectives are to evaluate the intervention’s effects on clinical outcomes such as postoperative pain, hip joint function recovery, quality of life, complication rates, and length of hospital stay. Based on these objectives, the following hypotheses are proposed: (1) Primary hypothesis: Elderly patients after femoral intertrochanteric fracture surgery who receive EBN intervention based on Watson’s theory will demonstrate better rehabilitation compliance than those receiving conventional care. (2) Secondary hypothesis: Compared to routine care, EBN intervention based on Watson’s theory will exhibit superior pain control, faster functional recovery, and higher quality of life. By validating these hypotheses, this study aims to provide new nursing ideas and intervention methods for postoperative rehabilitation in elderly patients with femoral intertrochanteric fractures, further promoting the application of EBN in the elderly patient population to improve rehabilitation quality and reduce family and societal burdens.

## Materials and methods

### Ethics statement

This study was approved by the Ethics Review Committee of The Affiliated Hospital of Xuzhou Medical University.

### Subjects of the study

**FIGURE 1:**
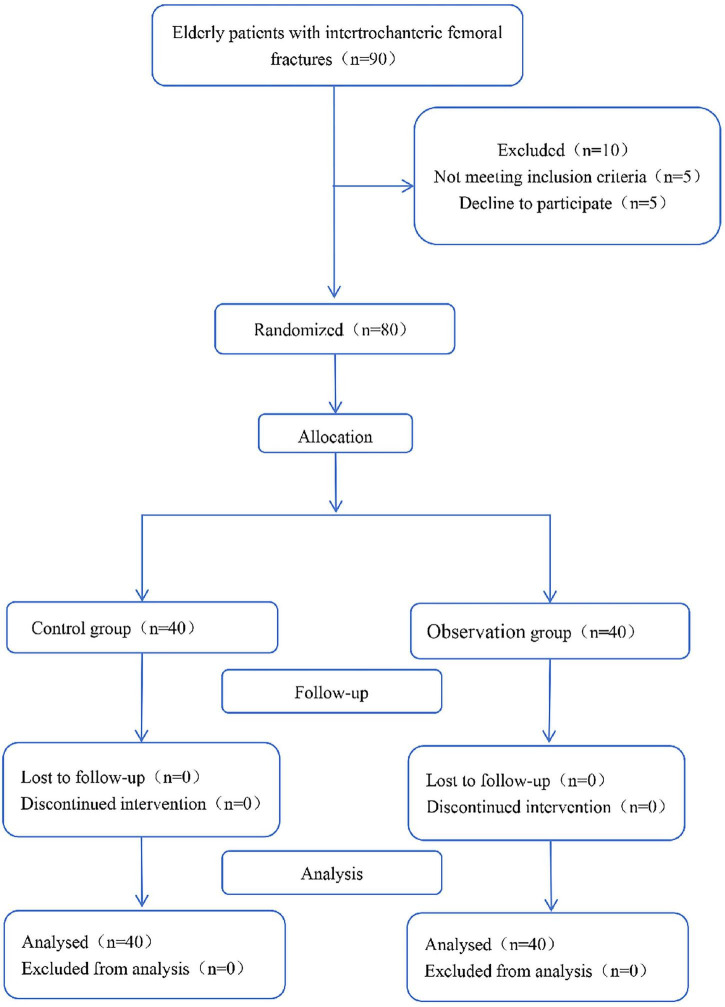
CONSORT flow diagram.

A prospective randomized controlled trial design was adopted. Ninety elderly patients with intertrochanteric femur fractures were selected, and ultimately, 80 cases were included (the CONSORT flow diagram is shown in Figure 1). Among them, there were 45 males and 35 females, aged 60–80 (69.96 ± 6.02) years old. All patients were randomly divided into a control group and an observation group, with 40 patients in each group. The control group was given routine intervention, and the observation group was given EBN based on Watson’s theory.

#### Inclusion criteria

Patients with a diagnosis of intertrochanteric femur fractures confirmed by X-ray/CT examination; patients who underwent proximal femur anti-rotation intramedullary nail fixation; patients with informed consent; patients with a certain degree of communication and comprehension; patients aged 60 years or older, who voluntarily participated in this study. All patients included in this study were operated on by the same orthopedic surgical team, which had rich experience in surgery for elderly intertrochanteric femur fractures. Fracture reduction and internal fixation were performed following unified surgical norms.

#### Exclusion criteria

Patients with severe lesions of the liver, kidneys, and other important organs; patients with acute and chronic infections; and patients with the presence of old fractures.

### Sample size calculation

The *post-hoc* power analysis was conducted using G*Power 3.1.9.7 software (University of Düsseldorf, Germany). In this study, an independent two-sample *t*-test was used. With a two-sided significance level of α = 0.05, based on the actually included sample size (40 cases in each of the two groups, with a total sample size of 80 cases) and a preset medium-to-large effect size (Cohen’s *d* = 0.7), the actual statistical power (1—β) of this study was calculated to be 0.81, meeting the conventional statistical test power requirement of 0.8.

### Randomization and blinding

The random number table method was used for grouping: The 80 study subjects were numbered from 1 to 80 according to the order of enrollment. Eighty corresponding random numbers were drawn from the Random Number Table. After arranging the random numbers in ascending order, the first 40 cases were included in the control group, and the last 40 cases were included in the observation group. The grouping plan was formulated and sealed for storage by an independent statistician. It was unsealed by non-study implementers after patient enrollment, and the researchers were unaware of the grouping results before the intervention implementation. Due to the particularity of the intervention measures, blinding of the intervention implementers was not feasible in this study. To minimize bias as much as possible, blinding was implemented for the outcome assessors, data inspectors, and statistical analysts in this study. All assessment materials were only marked with patient numbers. Subjective outcome indicators were filled out independently by the patients and then submitted in a sealed manner. The assessors and analysts only handled anonymous data throughout the process and had no access to information about patient grouping and interventions.

### Intervention methods

The control group was given routine nursing interventions: Upon admission, a comprehensive assessment of the patient’s overall condition and disease status was immediately completed. Based on the assessment results, targeted dietary guidance, standardized medication management, and stepwise activity guidance were provided. The risk of postoperative complications was dynamically monitored throughout the process, and timely measures such as analgesic interventions and standardized skin care were taken. In addition, routine psychological counseling and health education were provided.

**FIGURE 2:**
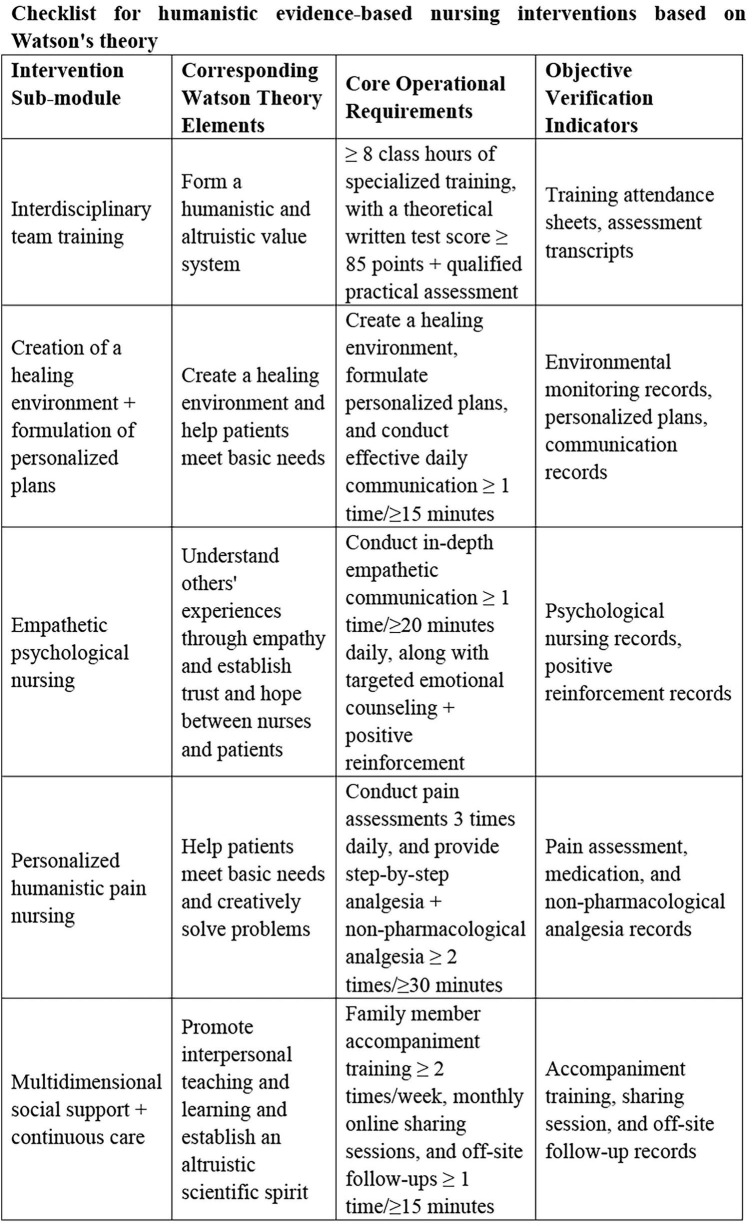
Checklist for humanistic evidence-based nursing interventions based on Watson’s theory.

The observation group received humanistic evidence-based nursing interventions based on Watson’s caring theory in addition to routine nursing. The intervention plan was centered around the ten caring elements of Watson’s theory and designed by integrating the evidence-based basis and clinical evidence from the “Expert Consensus on Perioperative Nursing for Elderly Orthopedic Patients in China” and the “Expert Consensus on Postoperative Pain Management in Adults.” Each measure was clearly corresponding to the elements of Watson’s theory, and standardized operational requirements and objective verification indicators were formulated, forming the “Checklist for humanistic evidence-based nursing interventions based on Watson’s theory” (Figure 2), which created a structural and standardized difference from conventional nursing. The specific measures are as follows:

➀ 【Forming a humanistic and altruistic value system】 An interdisciplinary Watson humanistic caring intervention team was established, including orthopedic attending physicians, clinical psychologists, and specialist nurses. A total of ≥ 8 class hours of specialized training were conducted, covering the core connotations of Watson’s theory, empathic communication skills, and standardization of the intervention process. After the training, a theoretical written test (a passing score of ≥ 85) + practical operation assessment were carried out, with a 100% passing rate. Those who failed were required to undergo retraining and retake the test until they passed, ensuring the unity of nursing concepts and operational norms, which was different from the conventional nursing implementation model without specialized training in the control group.
➁ 【Creating a healing environment and helping patients meet basic needs】 A healing inpatient environment was created to foster a warm and strong atmosphere of humanistic care. Based on Watson’s concept of “holistic care,” personalized nursing intervention plans were formulated with the patient’s fracture type, age, comorbidities, personality traits, and pain tolerance as the core evaluation criteria. An adaptive communication method was used to establish an effective connection with the patients (speaking slowly and using gestures for the elderly/those with hearing loss, listening patiently and providing gentle guidance for introverted patients). Effective communication was carried out at least once a day for a duration of ≥ 15 min, strengthening respect and emotional care for elderly patients. Through playing rehabilitation demonstration videos and conducting on-site movement demonstrations, patients were helped to master the essentials of rehabilitation exercises. At the same time, patients were guided to actively participate in the formulation of training plans to improve their compliance, which was different from the conventional health education in the control group without personalized plans and adaptive communication.
➂ 【Understanding others’ experiences through empathy and establishing trust and hope between nurses and patients】 Watson’s theory-oriented empathic psychological nursing was implemented: A deep empathic communication session lasting ≥ 20 min was conducted once a day. The nurses communicated deeply with the patients in a kind and patient manner, accurately capturing the patients’ emotional states and psychological needs to establish a trusting nurse-patient relationship. Empathy was used to understand the patients’ negative emotions such as fear of surgery and anxiety about rehabilitation, encouraging the patients to fully express their inner feelings and providing targeted emotional counseling. Through positive incentives and affirmation of rehabilitation progress, patients were helped to build confidence in rehabilitation, strengthen self-worth identification, and guide them to return to a positive psychological state, which was different from the conventional psychological counseling in the control group without a fixed process and deep empathy.
➃ 【Helping patients meet basic needs and creatively solving problems】 Personalized humanistic pain nursing was carried out: The patient’s pain level was regularly assessed and the chief complaints were recorded every morning, afternoon, and before bedtime. Stepwise analgesic interventions were implemented according to the evidence-based guidelines for postoperative pain management in orthopedics, and the changes in pain scores and medication reactions were accurately recorded. At the same time, non-pharmacological analgesic methods were used according to the patient’s preferences (accompanying the patient in chatting, playing film and television programs, and listening to soothing music) twice a day for 30 min each time to distract the patient’s attention, relieve pain perception, and improve comfort, which was different from the conventional pain nursing in the control group with on-demand analgesia and no standardized process for non-pharmacological analgesia.
➄ 【Promoting interpersonal teaching and learning and establishing an altruistic scientific spirit】 A multi-dimensional social support care system was constructed: Family members were instructed to accompany the patients in conducting rehabilitation training at least twice a week, with a 100% completion rate of records. Online rehabilitation experience sharing sessions were organized once a month, inviting patients with good rehabilitation to share their experiences and exert a peer support effect. An off-site continuous intervention mechanism was established. After the patients were discharged from the hospital, they were followed up once a week for at least 15 min each time via telephone/WeChat video calls, with a 100% timely follow-up rate and completion rate of records. The home rehabilitation situation was dynamically understood, questions were answered, and patients were urged to perform home rehabilitation exercises, achieving continuous care inside and outside the hospital, which was different from the conventional nursing model in the control group without systematic social support and standardized off-site follow-up.

Both groups were intervened by nurses with at least 5 years of clinical experience in orthopedics, and the intervention period was 3 months for both groups. In the observation group, all team members completed specialized training and assessment on Watson’s theory and evidence-based intervention processes before the intervention, and intervention fidelity checks were conducted once a week during the intervention process to ensure the standardized and normalized implementation of intervention measures. There were no relevant training and check requirements in the control group.

### Observation indicators and evaluation criteria

The Visual Analogue Scale (VAS) (12) was executed to observe the pain of the two groups at 3 day, 7 day, and 1 month postoperatively. By drawing a 10 cm horizontal line on the paper, one end is 0 to indicate no pain, and the other end is 10 to indicate acute pain. A point was marked by patients representing their own pain intensity, and the distance between the pain-free point (0) and the patient’s marker was measured with a ruler to determine the score.

Postoperative weight-bearing walking time, fracture healing time, and hospitalization time were compared between the two groups. The weight-bearing walking time is the time required for the patient to be able to walk with weight from the beginning of the surgery, the fracture healing time is the time from the end of the surgery to the healing of the fracture, and the hospitalization time is the time from the patient’s admission to the hospital to the discharge.

The World Health Organization quality of life-BREF (WHOQOL-BREF) (13) was implemented to evaluate the quality of life before and after 3 months of intervention in both groups. The scale consists of 26 items in 4 dimensions (physiological, psychological, environmental, and social relations) with a total score of 140. The higher the score, the higher the quality of life.

A self-made “Questionnaire on Rehabilitation Compliance after Surgery for Elderly intertrochanteric femur fractures” was used for assessment. The questionnaire was designed around five core dimensions: training content, training time, training intensity, initiative in rehabilitation training, and movement completion. Compliance was divided into three levels: complete compliance, partial compliance, and non-compliance. The rehabilitation compliance rate = (number of completely compliant cases + number of partially compliant cases)/total number of cases × 100%. This questionnaire was reviewed and revised by two orthopedic nursing experts, and its reliability and validity were verified in a pre-experiment (Cronbach’s α coefficient of 0.85 and scale-level content validity index S-CVI of 0.90), both reaching the acceptable standards for clinical research.

A self-made “Questionnaire on Nursing Satisfaction after Surgery for Elderly intertrochanteric femur fractures” was used to assess patient’s nursing satisfaction 3 months after the intervention. This questionnaire was also reviewed and revised by two orthopedic nursing experts, and its reliability and validity were verified in a pre-experiment (Cronbach’s α coefficient of 0.86 and scale-level content validity index of 0.92). The questionnaire divided satisfaction into three levels: very satisfied, satisfied, and dissatisfied. Satisfaction = (number of very satisfied cases + number of satisfied cases)/total number of cases × 100%. During the assessment, the patients independently filled out the scale and submitted it in a sealed manner to the blinded assessors, who only scored it based on the patient’s anonymous numbers and were unaware of the patients’ grouping and intervention plans throughout the process.

The Harris hip score (HHS) (14) was employed to evaluate the recovery of joint function before nursing and after 3 months of nursing in both groups. The HHS includes pain, function, functional activity, deformity, and mobility. A total of 10 items were included, with a total score of 100. The higher the score, the better the hip function.

The incidence of adverse reactions or complications, including joint stiffness, pressure ulcers, and deep venous thrombosis of the lower extremities, was compared between the two groups, and the overall incidence was calculated.

### Statistical analysis

All data were analyzed using SPSS 25.0 software (SPSS Inc, Chicago, IL, United States). For categorical data, the Chi-square test was applied, and percentages (%) were used for description. For continuous data, normal distribution was tested using the Shapiro-Wilk test. When the data followed a normal distribution, they were described as mean ± standard deviation; when the data did not follow a normal distribution, they were described using median (interquartile range). Levene’s test was adopted to assess homogeneity of variance. For normally distributed data with equal variance, independent sample *t*-tests were adopted for inter-group comparisons; otherwise, the Wilcoxon signed-rank test was applied. The difference of *P* ≤ 0.05 was statistically significant.

## Results

### Baseline data

The control group included 23 males and 17 females; the age ranged from 60 to 80 years, with an average age of (70.00 ± 5.55) years. Disease type: 6 cases of type I fracture, 13 cases of type II fracture, 11 cases of type III fracture, and 10 cases of type IV fracture. The observation group included 22 males and 18 females; the age ranged from 60 to 80 years, with an average age of (69.93 ± 6.52) years. Disease type: 5 cases of type I fracture, 12 cases of type II fracture, 13 cases of type III fracture, and 10 cases of type IV fracture. There was no statistically significant difference in the baseline data between the two groups (*P* > 0.05) (Table 1).

**TABLE 1:** Comparison of general patient information between the two groups (*n* = 40).

Grouping	Control group	Observation group	*T*-value/χ ^2^-value	*P*-value
Gender ratio (cases)			0.051	0.822
Male	23 (57.50)	22 (55.00)		
Female	17 (42.50)	18 (45.00)		
Age (years)	70.00 ± 5.55	69.93 ± 6.52	−0.237	0.813
*Disease type [n (%)]			0.298	0.960
Type I fracture	6 (15.00)	5 (12.50)		
Type II fracture	13 (32.50)	12 (30.00)		
Type III fracture	11 (27.50)	13 (32.50)		
Type IV fracture	10 (25.00)	10 (25.00)		

*According to the Evans-Jensen classification (28), it is divided into types I—IV. Type I: Simple intertrochanteric fracture without displacement, with an intact calcar femorale and stable fracture end; Type II: Intertrochanteric fracture with mild displacement, with a lesser trochanter fracture but an intact calcar femorale and relatively stable; Type III: Intertrochanteric fracture with significant displacement, with a lesser trochanter fragmentation, a calcar femorale fracture, and a greater trochanter fracture, and poor stability; Type IV: Reverse intertrochanteric fracture, with an opposite fracture line direction and significant displacement, being an unstable fracture.

### VAS scores

The VAS pain scores at 3 days, 7 days, and 1 month after surgery were compared. The VAS scores at 3 days, 7 days, and 1 month after surgery in the observation group were remarkably lower than those in the control group (*P* < 0.01), indicating that EBN based on Watson’s theory can reduce postoperative pain in patients (Table 2).

**TABLE 2:** Comparison of VAS scores between the two groups (*n* = 40).

Grouping	VAS score 3 days post-operative	VAS score 7 days post-operative	Post-operative 1-month VAS score
Control group	6.98 ± 1.30	6.01 ± 1.61	2.74 ± 1.69
Observation group	6.64 ± 1.56	5.06 ± 1.93	2.06 ± 1.31
*t*-value	−1.487	2.35	−2.025
*P*-value	0.137	0.020	0.043

### Rehabilitation indicators

The postoperative weight-bearing walking time, fracture healing time, and length of hospital stay were compared between the two groups. Better rehabilitation indicators were noted in the observation group (*P* < 0.01), suggesting that EBN based on Watson’s theory can promote patient recovery (Table 3).

**TABLE 3:** Comparison of rehabilitation indicators between the two groups (*n* = 40).

Grouping	Weight-bearing walking time (d)	Fracture healing time (d)	Length of hospital stay (d)
Control group	82.56 ± 10.22	70.06 ± 3.76	20.07 ± 4.97
Observation group	40.63 ± 8.90	37.56 ± 3.67	10.86 ± 4.66
*t*-value	19.505	38.641	8.514
*P*-value	<0.001	< 0.001	<0.001

### WHOQOL-BREF score

The WHOQOL-BREF scores of the two groups before and 3 months after the intervention were compared. The WHOQOL-BREF scores of both groups were notably increased after the intervention (*P* < 0.01) compared with before the intervention. After the intervention, the higher WHOQOL-BREF scores were noted in the observation group (*P* < 0.01), indicating that EBN based on Watson’s theory can improve patients’ quality of life (Table 4).

**TABLE 4:** Comparison of WHOQOL-BREF scores between the two groups (*n* = 40).

Grouping	Pre-intervention	After 3 months	*t*-value	*P*-value
Control group	67.14 ± 10.64	88.48 ± 8.45	−10.1	<0.001
Observation group	67.24 ± 10.76	118.69 ± 8.49	−22.04	<0.001
*t*-value	−0.01	−15.87		
*P*-value	0.99	<0.001		

### Rehabilitation compliance

The rehabilitation compliance rate in the observation group was 90%, which was noticeably higher relative to the control group (*P* < 0.01). This suggests that EBN based on Watson’s theory can improve rehabilitation compliance (Table 5).

**TABLE 5:** Comparison of rehabilitation compliance between the two groups (*n* = 40).

Grouping	Completely compliant	Partially compliant	Non-compliance	Compliance rate
Control group	5 (12.50)	10 (25.00)	25 (62.50)	15 (37.50)
Observation group	27 (67.50)	9 (22.50)	4 (10.00)	36 (90.00)
χ^2^ value				30.39
*P*-value				< 0.001

### Satisfaction

There exhibited improved satisfaction in the observation group versus the control group (*P* < 0.01), indicating that Watson’s theory-based EBN can increase satisfaction (Table 6).

**TABLE 6:** Comparison of satisfaction between the two groups (*n* = 40).

Grouping	Very satisfied	Satisfactory	Unsatisfactory	Satisfaction rate
Control group	8 (20.00)	9 (22.50)	23 (57.50)	17 (42.50)
Observation group	27 (67.50)	10 (25.00)	3 (7.50)	37 (92.50)
χ^2^ value				25.75
*P*-value				<0.001

### HHS

HHS was compared between the two groups before and 3 months after the intervention. Both groups exhibited elevated HHS after the intervention (*P* < 0.01). After 3 months of intervention, the observation group showed higher HHS (*P* < 0.01), indicating that EBN based on Watson’s theory can improve joint function (Table 7).

**TABLE 7:** Comparison of Harris hip scores between the two groups (*n* = 40).

Grouping	Before intervention	After 3 months of intervention	*t*-value	*P*-value
Control group	60.08 ± 9.28	75.74 ± 9.10	−7.81	<0.001
Observation group	60.66 ± 9.44	88.32 ± 7.58	−16.20	<0.001
*t*-value	−0.02	5.35		
*P-*value	0.99	< 0.001		

### Adverse reactions or complications

Watson’s theory-based EBN effectively reduced adverse reactions and complications in patients with intertrochanteric femur fractures (χ^2^ = 6.275, *P* = 0.012) (Table 8).

**TABLE 8:** Comparison of adverse reactions or complications between the two groups (*n* = 40).

Grouping	Joint stiffness	Pressure sores	Deep venous thrombosis	Incidence
Control group	5 (12.50)	3 (7.50)	2 (5.00)	10 (25.00)
Observation group	2 (5.00)	0	0	2 (5.00)
χ^2^ value				6.275
*P*-value				0.012

## Discussion

Intertrochanteric femur fractures are the most prevalent fractures among the elderly, often resulting in high rates of morbidity, mortality, and a diminished quality of life (15). In this study, EBN intervention based on Watson’s theory has a positive effect on enhancing postoperative rehabilitation compliance, alleviating postoperative pain at different stages, promoting hip joint function recovery, shortening the rehabilitation process, reducing complication risks, and improving the quality of life in elderly patients with intertrochanteric femur fractures.

First, the results of this study showed that the VAS scores of the observation group at 3 days, 7 days, and 1 month postoperatively were significantly lower than those of the control group. This finding is consistent with the principles of caring and compassion emphasized in Watson’s theory of nursing, which advocates that caregivers understand the patient’s pain and needs by connecting with the patient emotionally, and then provide personalized interventions during the nursing process. Besides, EBN has proven effective in minimizing postoperative complications and alleviating pain in patients undergoing subtotal gastrectomy for gastric cancer (16). This approach helps to alleviate patients’ postoperative distress, thereby reducing pain perception and promoting psychological recovery. Carpenter et al. “s study, guided by Watson”s theory of human caring, systematically evaluated the application value of mind-body interventions in postoperative pain management in orthopedics, pointing out that the holistic nursing concept advocated by Watson’s theory can integrate physical, psychological, and spiritual interventions to provide comprehensive pain management for postoperative patients (17). Based on the findings of this study, EBN intervention based on Watson’s theory has a positive effect on alleviating patient pain in the middle and late postoperative periods.

In a previous study, EBN has demonstrated strong clinical utility in managing pain in advanced lung cancer, enhancing patients’ sleep, quality of life post-treatment, and nursing satisfaction. It is recommended for widespread clinical implementation (18). The results of this study also demonstrated the effectiveness of EBN intervention based on Watson’s theory in shortening the postoperative rehabilitation cycle, promoting early functional recovery, reducing hospital stay, improving postoperative quality of life (higher WHOQOL-BREF scores in the observation group), and facilitating joint function recovery (higher Harris hip scores in the observation group). A study titled “Palliative Care Based on Watson’s Theory of Human Caring” emphasized the applicability of this framework in meeting patients’ diverse physical, social, and spiritual needs, indicating that a patient-centered holistic approach can enhance overall wellbeing (19). Similar to the findings of this study, Wen et al. ‘s research reported that after nursing intervention based on Watson’s theory, patients’ scores in all dimensions of the WHOQOL-BREF (physiological, psychological, environmental, and social relationships) and the excellent rate of Harris hip scores improve (20). This study clearly pointed out that Watson’s theory can systematically improve the quality of life of postoperative fracture patients by focusing on their overall needs (including physiological comfort, psychological support, and social interaction) (20).

An important finding of this study was the significant increase in rehabilitation compliance rate in the observation group compared to the control group, suggesting that EBN intervention based on Watson’s theory can improve patients’ cooperation in rehabilitation training. Compared to traditional methods that mainly rely on health education and supervision to enhance compliance, interventions based on Watson’s theory fundamentally stimulate patients’ intrinsic rehabilitation motivation by establishing a “helping-trusting” relationship, fostering hope, and respecting individual choices (7, 9, 21). This may be the key mechanism underlying the significant improvement in compliance rate. It has been reported that EBN intervention can improve treatment compliance in lung carcinoma patients receiving radiotherapy and chemotherapy (22). Another report indicated that the combination of Watson’s theory of human caring and respiratory techniques is significant for improving the nurse-patient relationship and building a harmonious hospital environment (23). Gönen relationship a ‘s study in hemodialysis patients also reported that support group interventions based on Watson’s theory significantly improve patients’ treatment compliance, including fluid intake restriction, dietary control, and medication management (10).

Notably, satisfaction was higher in the observation group, and the incidence of adverse events was lower in the observation group. Existing evidence supports that EBN implementation in patients with vertebral osteoporotic fractures improves postoperative activities of daily living, reduces the incidence of postoperative complications, and enhances patient satisfaction with nursing care (24). It has been reported that EBN intervention can alleviate fear of postoperative rehabilitation in elderly patients undergoing hip fracture surgery, improving rehabilitation treatment compliance and patient self-efficacy (25). A health promotion program based on Watson’s theory of human caring facilitated sustained self and holistic care and improved consistency and wellbeing among patients and caregivers (9).

From a mechanistic perspective, on the one hand, Watson’s theory of human caring alleviates anxiety and kinesiophobia in elderly patients by establishing a “helping-trusting” nurse-patient relationship, enhancing self-efficacy, thereby stimulating intrinsic rehabilitation motivation and improving rehabilitation compliance (10, 17). Through holistic care, it integrates physiological, psychological, and social needs to systematically improve patients’ postoperative quality of life (26). On the other hand, EBN provides standardized operational guidelines for key nursing aspects such as pain management, early mobilization protocols, and complication prevention by systematically retrieving and screening the best clinical evidence, ensuring the scientific and effective nature of nursing measures (27). These two aspects may form a synergistic model of “theory-driven motivation + evidence-guided practice”: Watson’s theory addresses the issue of patients’ “willingness to do” (stimulating intrinsic motivation), while EBN addresses the issues of “what to do and how to do it” (providing scientific protocols), jointly promoting significant improvements in multiple clinical outcomes in the observation group of this study.

The strengths of this study lie in its prospective randomized controlled trial design, which effectively controls selection bias to a certain extent. The outcome measures cover both subjective and objective indicators, including pain (VAS), function (Harris), quality of life (WHOQOL-BREF), rehabilitation compliance, complications, and length of hospital stay, validating the intervention effects from multiple perspectives. The intervention protocol is conceptualized within the framework of Watson’s theory of human caring rather than empirically piecing together nursing measures, enhancing the theoretical interpretability and reproducibility of the study. Additionally, the study specifically targets elderly patients with femoral intertrochanteric fractures, a population whose rehabilitation compliance issues pose clinical nursing challenges, making the results directly clinically instructive.

However, this study also has some limitations. Firstly, bundling multiple nursing measures guided by Watson’s theory for implementation makes it impossible to distinguish the independent contributions of each component. Future studies could adopt a factorial design to identify core active ingredients. Furthermore, potential confounding factors such as cognitive function, education level, and family support systems in elderly patients were not included in stratified analysis or covariate adjustment. Thirdly, the single-center design limits the generalizability of the results, and the nursing team’s proficiency in Watson’s theory may exhibit institutional specificity. Fourthly, the limited follow-up period prevents assessment of the impact on long-term outcomes such as secondary fracture rates and long-term functional independence.

In conclusion, systematically integrating Watson’s theory of human caring into the EBN framework to construct a nursing intervention model plays a crucial role in enhancing patients’ intrinsic rehabilitation motivation and compliance, promoting functional recovery, improving quality of life, and potentially reducing complication risks after femoral intertrochanteric fracture surgery in the elderly. The results of this study preliminarily validate the operational feasibility of this integrated model. The core innovation and theoretical contribution of this study lie in the first construction and validation of an integrated intervention model of “EBN based on Watson”s “theory of human caring” and its application in elderly patients after femoral intertrochanteric fracture surgery. This differs from traditional EBN interventions that focus on standardized processes and physiological outcomes. Based on the limitations of this study, future research should conduct multicenter randomized controlled trials to validate the generalizability of the results; extend the follow-up period to assess long-term functional prognoses. Additionally, factorial designs could be considered to identify core active ingredients; explore potential physiological-psychological mediating mechanisms (such as stress hormones, neuroendocrine indicators). Furthermore, training courses based on Watson’s theory could be developed to promote the implementation of this model in clinical nursing.

## Data Availability

The original contributions presented in the study are included in the article/supplementary material, further inquiries can be directed to the corresponding author.
